# Salivary proteome signatures in the early and middle stages of human pregnancy with term birth outcome

**DOI:** 10.1038/s41598-020-64483-6

**Published:** 2020-05-15

**Authors:** Amit Kumar Dey, Bhoj Kumar, Abhishek Kumar Singh, Prakash Ranjan, Ramachandran Thiruvengadam, Bapu Koundinya Desiraju, Pallavi Kshetrapal, Nitya Wadhwa, Shinjini Bhatnagar, Faraz Rashid, Dipankar Malakar, Dinakar M. Salunke, Tushar Kanti Maiti, GARBH-Ini Study Group*, Bhabatosh Das, Sumit Misra, Balakrish G. Nair, Uma Chandra Mouli Natchu, Satyajit Rath, Kanika Sachdeva, Shailaja Sopory, Amanpreet Singh, Dharmendra Sharma, Vineeta Bal, Arindam Maitra, Partha P. Majumder, Monika Bahl, Sunita Sharma, Umesh Mehta, Brahmdeep Sindhu, Sugandha Arya, Rekha Bharti, Harish Chellani, Pratima Mittal, Siddarth Ramji, Reva Tripathi, Anju Garg, Ashok Khurana, Smriti Hari, Yashdeep Gupta, Nikhil Tandon, Rakesh Gupta

**Affiliations:** 1 Regional Centre for Biotechnology, NCR Biotech Science Cluster, Faridabad, 121001 India; 2 Translational Health Science and Technology Institute, NCR Biotech Science Cluster, Faridabad, 121001 India; 3 Sciex, 121 UdyogVihar, Gurgaon, Haryana 122015 India; 4 International Centre for Genetic Engineering and Biotechnology, Aruna Asaf Ali Marg, New Delhi, 110067 India; 5 Indian Institute of Science Education and Research, Pune, Maharashtra India; 6 National Institute of Biomedical Genomics, Kalyani, West Bengal India; 7 Clinical Development Services Agency, Translational Health Science and Technology Institute, NCR Biotech Cluster, Faridabad, Delhi NCR India; 8 Gurugram Civil Hospital, Gurugram, Haryana India; 9 Safdarjung Hospital, New Delhi, India; 10 Maulana Azad Medical College, New Delhi, India; 11 Hamdard Institute of Medical Sciences and Research, Jamia Hamdard University, New Delhi, India; 12 The Ultrasound Lab, Defence Colony, New Delhi, India; 13 All India Institute of Medical Sciences, New Delhi, India; 14 Government of Haryana, chandigarh, India

**Keywords:** Prognostic markers, Proteomic analysis

## Abstract

The establishment and maintenance of pregnancy in humans proceed through a continuous change of biochemical and biophysical processes. It requires a constant interaction between the fetus and the maternal system. The present prospective study aims to elucidate changes in salivary proteome from the early to middle stages of term pregnancy, and establishing an expressional trajectory for modulated proteins. To date, a comprehensive characterization of the longitudinal salivary proteome in pregnancy has not been performed and it is our immediate interest. In the discovery phase, maternal saliva (N = 20) at 6–13, 18–21, and 26–29 weeks of gestation was analyzed using level-free proteomics (SWATH-MS) approach. The expression levels of 65 proteins were found to change significantly with gestational age and distributed into two distinct clusters with a unique expression trajectory. The results revealed that altered proteins are involved in maternal immune modulation, metabolism, and host defense mechanism. Further, verification of 12 proteins was employed using targeted mass spectrometry (MRM-MS) in a separate subset of saliva (N = 14). The MRM results of 12 selected proteins confirmed a similar expression pattern as in SWATH-MS analysis. Overall, the results not only demonstrate the longitudinal maternal saliva proteome for the first time but also set the groundwork for comparative analysis between term birth and adverse pregnancy outcomes.

## Introduction

Human pregnancy and parturition are complex biological processes that proceed through the coordinated action of many biochemical signaling networks. There are many pieces of evidence to suggest that normal pregnancy induces transient physiological, hormonal, and immunological changes in a highly controlled and coordinated manner^1,2^. The maternal immune system plays a crucial role in maintaining the balance to tolerate fetal allograft while preserving innate and adaptive immune mechanisms for protection against microbial challenges^3,4^. Disturbance of the delicate balance of the biological processes leads to adverse pregnancy outcomes, one being preterm birth. Immune dysregulation due to microbial pathogenesis is also increasingly appreciated in preterm birth and other pregnancy-related complications^5,6^.

Body fluids like blood, saliva, tears, sweat, urine, and cerebrospinal fluid are a source of putative biochemical markers that reflect various pathophysiological disorders. The saliva secreted from salivary glands and gingival crevice has some components that originate from the plasma with an overlap of almost 20–30% with plasma proteome, and the majority of these exhibit antimicrobial activity, transport, and enzymatic functions^7,8^. Human saliva, therefore, is a potential diagnostic fluid that can reflect many pathophysiological states of the body^9^. The decrease in salivary pH, calcium, glucose and an increase in phosphate levels throughout pregnancy favor gingival inflammation and oral infections, which could have some associations with adverse pregnancy outcomes^10-16^. Extensive plasma proteomics has been studied in pregnancy and pregnancy-related complications in an attempt to find potential biomarkers^17–19^. However, a few saliva specific proteomics studies have been undertaken for pregnancy. It has been shown that the level of salivary placental growth factor (PlGF) is significantly lower in preeclampsia conditions than normal pregnancy^20^. Similarly, the level of anti-inflammatory protein Annexin-1 in the saliva is shown to be elevated in pregnant women with gingivitis^21^. Recent 2D-gel based-proteomics analysis of saliva from pregnant women who deliver premature babies has identified a Metallothionein 2 (MT2A) protein as a potential marker for preterm birth prediction^22^.

The present study demonstrates longitudinal maternal saliva proteome changes in normal pregnancy for the first time. The SWATH-MS analysis quantifies 65 proteins that show temporal variations with gestational age. These proteins are distributed into two distinct clusters with a unique expression trajectory. Neutrophil degranulation, antimicrobial peptide function, regulation of TLR by an endogenous ligand, platelet function regulation, and glucose metabolism are the major pathways associated with these proteins. The MRM-MS analysis of 12 selected proteins confirms a similar expression pattern as observed in discovery analysis. The proteomics study of saliva in term birth pregnancy cannot be derived in adverse pregnancy outcomes. However, this information may provide useful background knowledge to target the specific molecular pathways in biomarker discovery.

## Results

### Clinical characteristics of the participants

Our 34 study participants who fulfilled the inclusion criteria for this study represent the semi-urban population of a northern state in India. The median age of the participants was 23 (interquartile range (IQR) 20, 23) years and 24 (IQR, 22, 25) years respectively for the discovery and the validation cohorts (Table 1). All subjects delivered at term and had a baby with appropriate body weight for age (Table 1). The median gestational age at delivery and the median birth weight for the study population is shown in Table 1. Other clinical and socioeconomic characteristics of the participants are described in Table 1.

**Table 1:** Clinical and demographic characteristics of the study population.

Characteristic	Discovery cohort (n = 20)	Validation cohort (n = 14)
Maternal age (years) (Median (IQR))	23 (20,23)	24 (22,25)
**Gestational age at biospecimens collection (weeks) (Median (IQR))**		
At enrolment (Visit 1)	10w3d (8w2d,12w5d)	11w3d (10w2d,12w4d)
At Visit 2	18w4d (18w1d,19w5d)	18w5d (18w3d,19w5d)
At Visit 3	26w3d (26w2d,27w4d)	26w2d (26w1d,26w3d)
BMI (kg/m^2^) (Median (IQR))		
At enrolment (Visit 1)	20.34 (18.69,22.37)	22.86 (19.31,25.18)
At Visit 2	21.34 (19.84,22.73)	23.14 (19.02,25.11)
At Visit 3	22.55 (20.56,24.10)	23.63 (21.08,26.80)
Parity (Median (IQR))	0 (0,1)	1 (0,1)
Gravida (Median (IQR))	2 (1,2)	2 (2,4)
**Socioeconomic status [n (%)]**		
Upper middle class	4 (20.00)	3 (21.43)
Lower middle class	9 (45.00)	4 (28.57)
Upper lower class	7 (35.00)	7 (50.00)
A habit of tobacco chewing [n (%)], Never	20 (100.00)	14 (100.00)
Gestational age at delivery (weeks) (Median (IQR))	38w3d (38w2d,39w5d)	40w3d (39w3d, 40w5d)
Birth weight (kg) (Median (IQR))	3.00 (2.65,3.11)	3.11 (2.91,3.28)
**Sex of the baby [n (%)]**		
Male	11 (55.00)	6 (42.86)
Female	9 (45.00)	8 (57.14)

### Differential proteomics of saliva with the progression of pregnancy in term delivery

**Figure 1:**
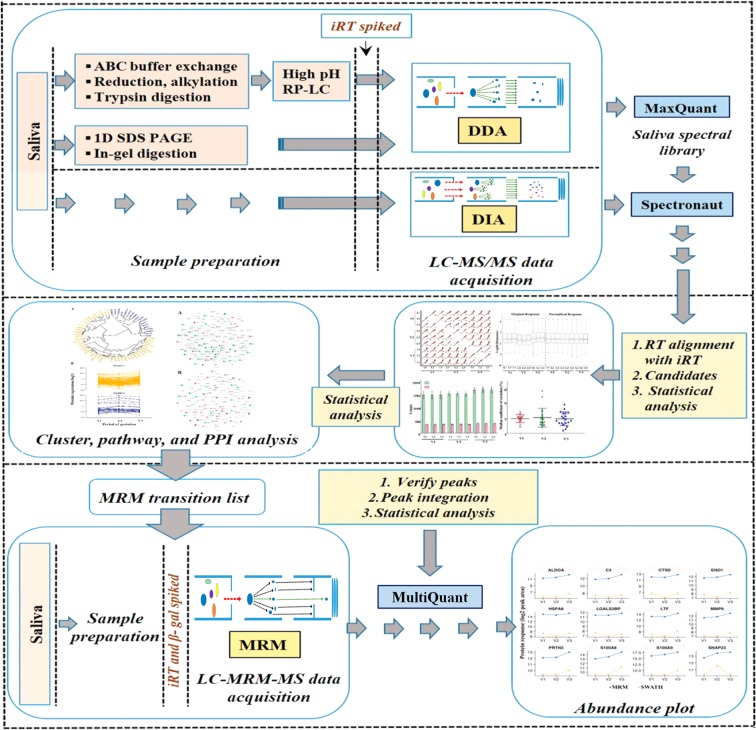
Schematic representation of the label-free quantitative discovery and MRM based targeted validation workflow. The label-free DIA approach with a saliva sample (N = 20) was taken to evaluate saliva proteome along with saliva-specific spectral library generation by DDA method. The DDA data were exported to MaxQuant for library generation and subsequently, each DIA file was analyzed with a prebuilt saliva spectral library in Spectronaut. An independent set of saliva samples (N = 14) was used to corroborate predicted central regulator proteins by MRM. More details can be found under Material and Methods.

**Figure 2:**
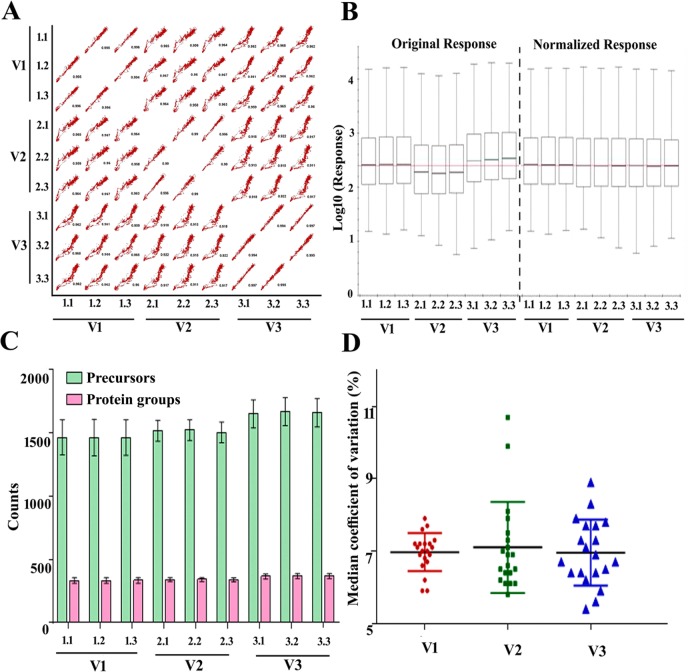
Quality assessment of label-free quantitative mass spectrometry data acquisition. (**A**) Scatter plot of peak intensities between the technical replicates of the salivary proteome of visit V1, V2, and V3, the Pearson correlation coefficient is indicated at the bottom of each plot. (**B**) The left side panel shows boxplots of precursor responses before normalization for each run at V1, V2, and V3 with technical repeats. Similarly, the right-side panel shows boxplots of the same precursor responses after normalization based on the median of total ion current (TIC). (**C**) Reproducibility of identification of the average precursors (green columns) and protein groups (pink columns) within the visit and technical repeats in all analyzed 20 saliva samples. Data represents mean ± SE of 20 independent data for each run at V1, V2, and V3 with technical repeats. (**D**) The dot plot shows the %CV distribution for all conditions, V1, V2, and V3. Data represent mean with SD of 20 independent data for each run at V1, V2, and V3.

The saliva-specific library was created by combining 1D SDS-PAGE followed by LC-MS/MS and bRP-C18-HPLC followed by LC-MS/MS for label-free quantitation of salivary proteins at different stages of pregnancy in SWATH-MS workflow (Fig. 1). MaxQuant analysis of combined LC-MS/MS library data yielded ~758 unique proteins in 1% FDR. The functional analysis of identified library proteins belonged to the immune system, metabolism, and signaling pathways (Supplementary Figure S1 and Table S1). The reproducibility in SWATH run between technical replicates (within triplicate) and within different time points (V1, V2, and V3) was uniform across all samples with average Pearson correlation value ~0.99 and ~0.95 respectively (Fig. 2A). The iRT peptides which were added in each sample, facilitated retention time (RT) normalization of all LC runs and ensured the specificity of fragment ion peak extraction^23^. However, to minimize the systematic variation all samples were normalized based on the median of total ion current (TIC) (Fig. 2B). The identification of precursor ions and protein groups within technical repeats was highly consistent in all samples, which confirms data reproducibility (Fig. 2C). The median coefficient of variance (CV) was 7, 7.1, and 6.9% over triplicate acquisition for all samples at V1, V2, and V3 time points, respectively (Fig. 2D). The data-independent acquisition (DIA) data resulted in the quantification of the average 2510 peptide precursors corresponding to ~446 protein groups with an average of 61% data set completeness across the time points in all 20 saliva samples. Sixty-five (15%; 65/446) proteins were modulated in abundance as a function of gestational age (p-value < 0.05; q-value < 0.1, Table 2).

**Table 2:** List of proteins modulates with the function of gestational age.

UniProt Id	Protein Name	Gene Name	FC(V2/V1)	FC(V3/V1)	FC(V3/V2)	p_values	q_values	Cluster
Q562R1	Beta-actin-like protein 2	ACTBL2	0.52	1.17	2.24	0.014	0.055	1
P43652	Afamin	AFM	1.29	2.02	1.56	0.000	0.000	1
P13716	Delta-aminolevulinic acid dehydratase	ALAD	0.78	1.19	1.53	0.022	0.078	1
P04075	Fructose-bisphosphate aldolase A	ALDOA	1.04	2.20	2.12	0.005	0.023	1
P19961	Alpha-amylase 2B	AMY2B	0.50	2.67	5.30	0.000	0.000	1
P01024	Complement C3	C3	0.72	1.10	1.52	0.011	0.045	1
P27797	Calreticulin	CALR	0.50	0.75	1.49	0.016	0.058	1
P08571	Monocyte differentiation antigen CD14	CD14	0.37	0.29	0.78	0.000	0.000	1
Q9NYQ6	Cadherin EGF LAG seven-pass G-type receptor 1	CELSR1	0.56	1.00	1.77	0.019	0.067	1
Q14019	Coactosin-like protein	COTL1	0.65	1.74	2.68	0.002	0.011	1
P00450	Ceruloplasmin	CP	0.73	1.10	1.50	0.005	0.022	1
P07339	Cathepsin D	CTSD	0.42	1.00	2.37	0.002	0.008	1
P81605	Dermcidin	DCD	0.56	1.15	2.03	0.012	0.046	1
P06733	Alpha-enolase	ENO1	0.67	1.43	2.14	0.003	0.013	1
P09104	Gamma-enolase	ENO2	0.68	1.21	1.78	0.000	0.002	1
P84090	Enhancer of rudimentary homolog	ERH	1.61	3.13	1.94	0.000	0.000	1
Q8N0U4	Protein FAM185A	FAM185A	0.72	1.06	1.47	0.021	0.075	1
P78417	Glutathione S-transferase omega-1	GSTO1	0.48	1.20	2.49	0.000	0.002	1
P16402	Histone H1.3	HIST1H1D	1.32	3.84	2.92	0.007	0.030	1
P11142	Heat shock cognate 71 kDa protein	HSPA8	0.49	1.10	2.23	0.000	0.000	1
P04792	Heat shock protein beta-1	HSPB1	1.03	3.59	3.47	0.014	0.053	1
P61604	10 kDa heat shock protein, mitochondrial	HSPE1	0.84	2.73	3.25	0.000	0.000	1
P01859	Immunoglobulin heavy constant gamma 2	IGHG2	0.49	0.65	1.31	0.011	0.044	1
A0A0C4DH38	Immunoglobulin heavy variable 5–51	IGHV5–51	0.30	0.58	1.93	0.000	0.000	1
A0A075B6K4	Immunoglobulin lambda variable 3–10	IGLV3–10	0.34	1.23	3.63	0.000	0.000	1
A0A0B4J1Y8	Immunoglobulin lambda variable 9–49	IGLV9–49	0.47	0.87	1.86	0.000	0.000	1
P07476	Involucrin	IVL	0.52	0.67	1.27	0.000	0.000	1
P04264	Keratin, type II cytoskeletal 1	KRT1	0.58	0.83	1.43	0.016	0.058	1
Q04695	Keratin, type I cytoskeletal 17	KRT17	0.24	1.38	5.83	0.000	0.000	1
P35908	Keratin, type II cytoskeletal 2 epidermal	KRT2	0.67	0.96	1.44	0.028	0.095	1
P35527	Keratin, type I cytoskeletal 9	KRT9	0.49	0.79	1.62	0.005	0.024	1
P13796	Plastin-2	LCP1	0.48	1.16	2.41	0.000	0.002	1
Q08380	Galectin-3-binding protein	LGALS3BP	0.60	0.80	1.33	0.001	0.003	1
P22079	Lactoperoxidase	LPO	0.84	1.61	1.92	0.000	0.001	1
P02788	Lactotransferrin	LTF	0.63	2.09	3.31	0.000	0.003	1
P40925	Malate dehydrogenase, cytoplasmic	MDH1	0.88	1.57	1.79	0.002	0.010	1
P14780	Matrix metalloproteinase-9	MMP9	0.98	1.67	1.72	0.004	0.019	1
P80303	Nucleobindin-2	NUCB2	0.37	0.85	2.29	0.001	0.008	1
P07237	Protein disulfide-isomerase	P4HB	0.69	1.14	1.64	0.010	0.039	1
Q6MZM9	Proline-rich protein 27	PRR27	0.70	1.33	1.91	0.019	0.067	1
P24158	Myeloblastin	PRTN3	1.06	1.64	1.54	0.002	0.009	1
P07602	Prosaposin	PSAP	0.32	1.35	4.22	0.000	0.000	1
P05109	Protein S100-A8	S100A8	0.91	3.14	3.46	0.000	0.001	1
P06702	Protein S100-A9	S100A9	0.91	2.97	3.26	0.000	0.000	1
P48595	Serpin B10	SERPINB10	0.54	1.17	2.14	0.000	0.000	1
P03973	Antileukoproteinase	SLPI	1.48	7.10	4.80	0.000	0.001	1
P57768	Sorting nexin-16	SNX16	0.36	0.87	2.45	0.000	0.000	1
Q92560	Ubiquitin carboxyl-terminal hydrolase BAP1	BAP1	3.89	2.67	0.69	0.000	0.000	2
P47755	F-actin-capping protein subunit alpha-2	CAPZA2	1.76	1.52	0.86	0.000	0.000	2
P16870	Carboxypeptidase E	CPE	0.76	0.52	0.68	0.004	0.018	2
P07711	Cathepsin L1	CTSL	0.79	0.77	0.97	0.004	0.017	2
Q16610	Extracellular matrix protein 1	ECM1	1.23	1.37	1.11	0.000	0.000	2
P24530	Endothelin receptor type B	EDNRB	1.37	0.49	0.35	0.003	0.015	2
P01877	Immunoglobulin heavy constant alpha 2	IGHA2	1.48	0.99	0.67	0.000	0.000	2
P01601	Immunoglobulin kappa variable 1D-16	IGKV1D-16	1.34	1.10	0.82	0.000	0.000	2
P01042	Kininogen-1	KNG1	1.06	0.99	0.94	0.000	0.000	2
P09382	Galectin-1	LGALS1	1.66	0.69	0.42	0.000	0.000	2
O95274	Ly6/PLAUR domain-containing protein 3	LYPD3	1.13	0.87	0.77	0.006	0.024	2
Q9NQR4	Omega-amidase NIT2	NIT2	1.09	0.89	0.82	0.000	0.000	2
Q99497	Protein/nucleic acid deglycase DJ-1	PARK7	0.95	1.00	1.05	0.000	0.000	2
Q9ULZ3	Apoptosis-associated speck-like protein containing a CARD	PYCARD	1.14	1.10	0.97	0.000	0.000	2
O00161	Synaptosomal-associated protein 23	SNAP23	2.35	0.95	0.40	0.017	0.060	2
P04004	Vitronectin	VTN	1.44	1.31	0.91	0.015	0.056	2
P62258	14–3–3 protein epsilon	YWHAE	3.23	1.75	0.54	0.000	0.000	2
Q9Y2Y4	Zinc finger and BTB domain-containing protein 32	ZBTB32	1.81	1.01	0.56	0.007	0.028	2

### Differentially regulated proteins constitute two distinct clusters

**Figure 3:**
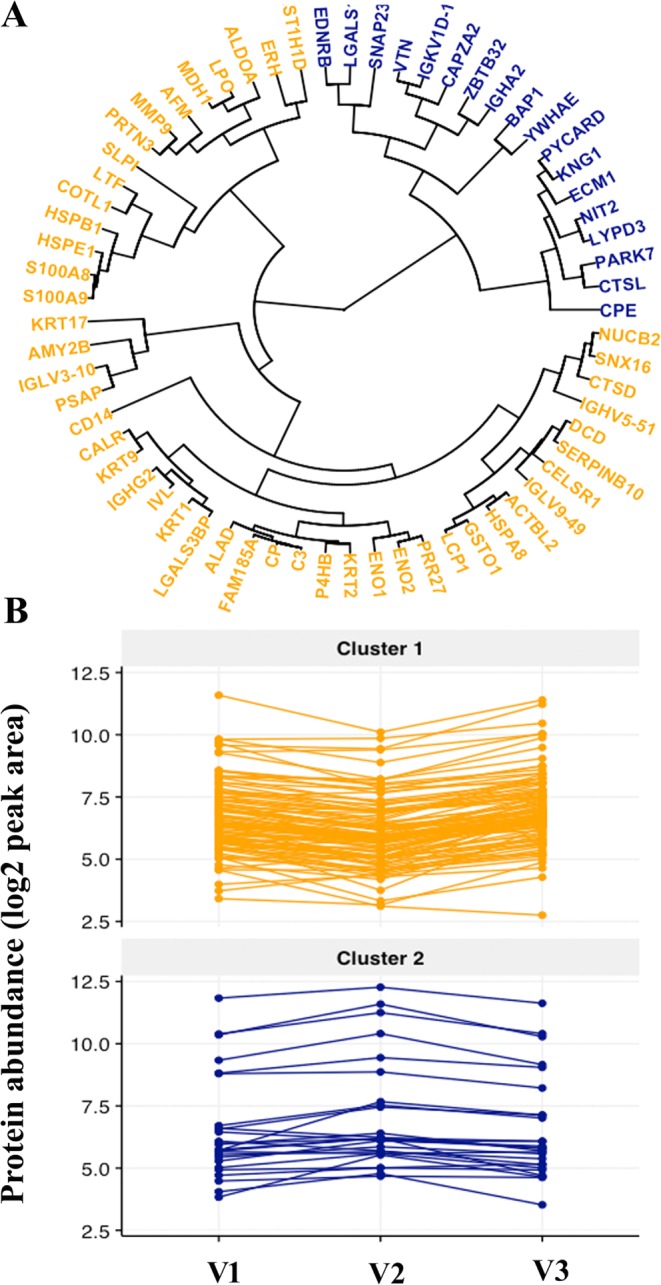
Hierarchical clustering and expressional trend within the cluster. A short time series was used for the construction of a hierarchical cluster. The circular plot of the dendrogram from the hierarchical clustering shows the presence of two clusters. The gene symbols in cluster 1 and cluster 2 are shown and are colored orange and blue respectively (**A**). The longitudinal profile of proteins across the pregnancy within two clusters 1 and 2. The line plot is showing protein abundance (log, base 2) on the y-axis and visit V1, V2, V3 respectively on the x-axis with each protein represented by a line (**B**). The top panel shows the proteins of cluster 1 (orange) and the bottom panel shows the proteins of cluster 2 (blue). The two clusters show the opposite trend with time.

Hierarchical with short time-series clustering of 65 modulated proteins resulted in two clusters derived from expression patterns across the gestational age (Fig. 3A). The clusters 1 and 2 were comprised of 47 and 18 proteins respectively. All proteins with their cluster possession and respective fold changes are shown in Table 2 and their expressional trajectory within a cluster is shown in Fig. 3B. The pathways enrichment by over-representation analysis of 65 proteins was performed. Overall 37 out of 65 proteins were significantly enriched in 15 biological pathways (p-value < 0.05, Table 3). The significant enrichment was observed within cluster1where 30 out of 37 proteins (81%) were from cluster 1 and rest 7 (19%) proteins were from cluster 2. Interestingly, 19 out of 37 (51%) were essentially enriched in neutrophil degranulation as the topmost pathway (Table 3). The rest of the 18 regulated proteins constituted 14 biological pathways which are listed in Table 3. The pathway analysis demonstrated that there was a significant overlap in biological processes between the two clusters. The proteins with their signature expression pattern within cluster 1 and 2 might support all the enriched pathways in a highly controlled and coordinated manner to maintain a healthy pregnancy.

**Table 3:** Enriched biological pathways linked with the proteins that are changed as a function of gestational age.

Biological pathways	Count	p-value	p.adjust	Gene Name	
				Cluster 1	Cluster 2
Neutrophil degranulation	19	0.000	0.000	C3, LTF, SLPI, ALDOA, KRT1, S100A8, S100A9, CTSD, PSAP, CD14, HSPA8, ALAD, MMP9, PRTN3, SERPINB10, COTL1	SNAP23, NIT2, PYCARD
Gluconeogenesis	4	0.000	0.002	ALDOA, ENO1, ENO2, MDH1	—
Regulation of TLR by endogenous ligand	3	0.000	0.005	S100A8, S100A9, CD14	—
Antimicrobial peptides	5	0.000	0.005	LTF, S100A8, S100A9, PRTN3, DCD	—
Post-translational protein phosphorylation	5	0.000	0.005	CP, C3, P4HB	KNG1, LGALS1
Regulation of Insulin-like Growth Factor (IGF) transport and uptake by Insulin-like Growth Factor Binding Proteins (IGFBPs)	5	0.000	0.008	CP, C3, P4HB	KNG1, LGALS1
Platelet degranulation	5	0.000	0.008	ALDOA, PSAP, LGALS3BP	KNG1, ECM1
Formation of the cornified envelope	5	0.000	0.008	KRT1, IVL, KRT9, KRT2, KRT17	—
Response to elevated platelet cytosolic Ca2+	5	0.000	0.008	ALDOA, PSAP, LGALS3BP	KNG1, ECM1
Glucose metabolism	4	0.001	0.016	ALDOA, ENO1, ENO2, MDH1	—
Antigen processing-Cross presentation	4	0.001	0.020	CD14, CALR	SNAP23, CTSL
Interleukin-12 signaling	3	0.001	0.025	P4HB, LCP1, GSTO1	—
Interleukin-12 family signaling	3	0.002	0.040	P4HB, LCP1, GSTO1	—
Keratinization	5	0.003	0.042	KRT1, IVL, KRT9, KRT2, KRT17	—
Collagen degradation	3	0.003	0.044	CTSD, MMP9	CTSL

### Protein-protein interaction (PPI) and network analysis identifies central regulators in pregnancy progression

**Figure 4:**
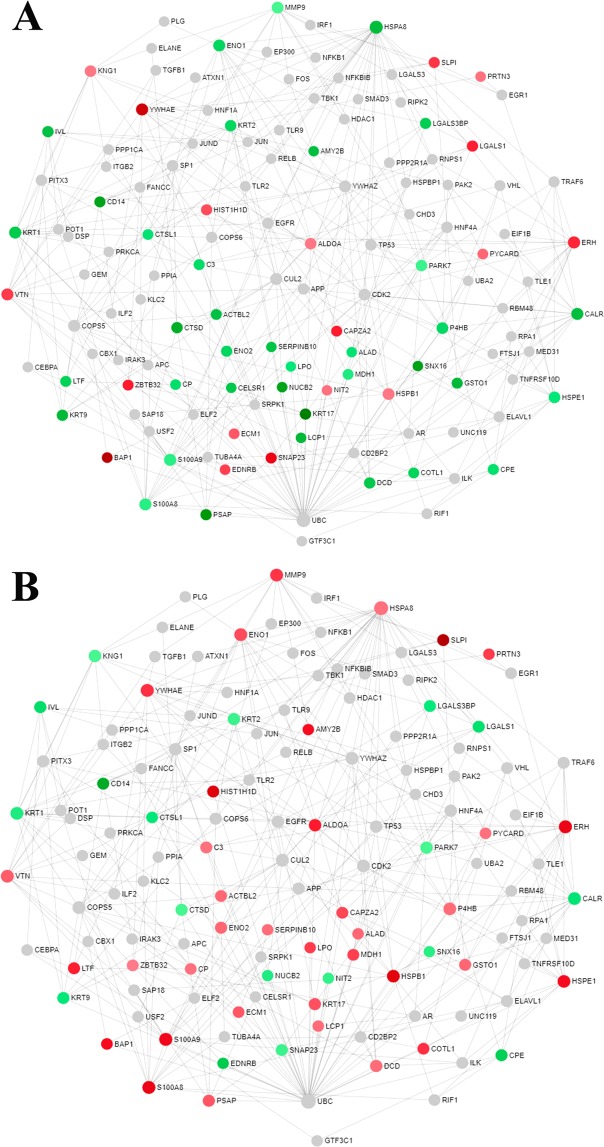
Protein-protein interaction network of modulated proteins to assess central regulators in the maintenance of normal pregnancy. The upper panel shows a ratio in protein abundance between visits, V2/V1 (**A**), and the lower panel shows between V3/V1 (**B**). “Minimum network” of the modulated proteins is shown in a force atlas layout format. Nodes represent individual proteins and edges between two nodes represent interactions between them. The colors denote the expressions of nodes. The red and green indicate up- and down-regulation, respectively. The intensity of the colors signifies expression levels.

Next, to investigate central regulatory proteins within saliva that hold the central network to maintain the normal pregnancy outcome, the PPI network was constructed with the expression profile of 65 proteins at V2 and V3 separately with respect to V1 (Fig. 4). The sub-network comprises of 129 nodes and 348 edges were formed with 55 seed proteins. Ten proteins were not included in this network. Interestingly, 67% (37/55) of proteins showed a downward trend (green circle) at V2, while 65% (36/55) demonstrated an upward trend (red circle) at V3 (Fig. 4A,B).

To identify central regulators within 65 proteins, network analysis of subnetwork was evaluated based on two parameters, degree centrality, and betweenness centrality. We identified 35 proteins whose degree centrality was at least 2 (Table 4). We considered these proteins as central regulators.

**Table 4:** Degree and betweenness centrality values of the central regulators.

UniProt	Gene Name	Degree	Betweenness
P11142	HSPA8	28	1397.52
P06702	S100A9	15	481.99
P27797	CALR	14	573.06
P05109	S100A8	14	404.06
P04264	KRT1	14	372.28
P06733	ENO1	13	376.47
P14780	MMP9	12	550.81
P07237	P4HB	11	250.29
P01042	KNG1	9	186.47
P35908	KRT2	8	186.79
P04075	ALDOA	8	51.04
P07339	CTSD	7	146.23
P07476	IVL	7	143.9
P35527	KRT9	7	49.75
P81605	DCD	7	44.03
P07602	PSAP	6	157.03
P09104	ENO2	6	89.57
Q08380	LGALS3BP	6	81.32
Q04695	KRT17	6	62.13
P09382	LGALS1	6	58.8
P01024	C3	5	104.42
P02788	LTF	5	100.55
P03973	SLPI	5	90.95
O00161	SNAP23	5	72.04
P08571	CD14	5	53.56
P78417	GSTO1	5	47.33
P07711	CTSL	4	95.39
Q14019	COTL1	4	41.35
P40925	MDH1	4	21.16
Q16610	ECM1	3	344.64
Q9ULZ3	PYCARD	3	16.95
P00450	CP	3	10.81
P13796	LCP1	2	21.26
P13716	ALAD	2	18.49
P24158	PRTN3	2	10.7

### Targeted validation of salivary proteins by MRM mass spectrometry

**Figure 5:**
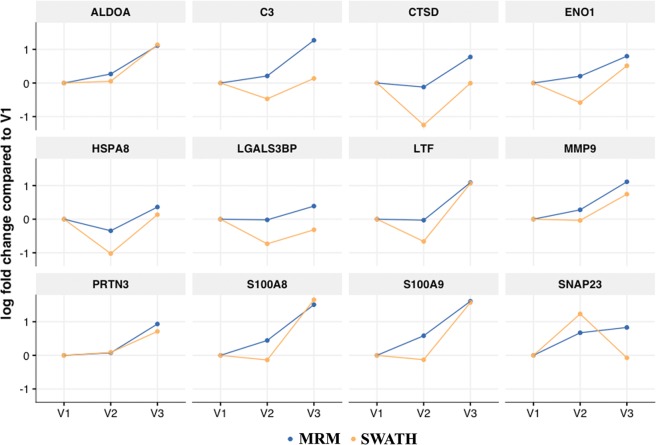
SWATH and MRM concurrence of temporal expression patterns of selected proteins. The x-axis of the figure represents the visits (V1, V2, and V3) and the y-axis represents a log2 of fold change with respect to V1. The mean of the proteins across samples at each visit is represented as a dot connected by lines and colored by the experimental technique.

The discovery phase label-free quantitation yielded 65 altered proteins of which 35 were assigned for central regulators. Here, twelve proteins had been selected based on their pathway enrichment performance (Table 3) for targeted label-free scheduled MRM based quantification in a separate verification cohort (N = 14). The transition assessment and peak integration showed consistency in retention time and peak area across all the samples. The retention time for β-galactosidase and iRT precursors was found consistent at visit window V1, V2, and V3, and within technical repeats with average CV < 2.0 (Supplementary Figure S2). A comprehensive list of precursors/fragments is given in Supplementary Table S3. Twelve proteins were modulated in abundance as a function of gestational age (p-value< 0.05; q-value < 0.1, Supplementary Table S4). Besides the expression trajectory of 12 proteins except for SNAP23 in MRM assay was showing a similar pattern to our discovery phase results (Fig. 5 and Table 2).

## Discussion

The current study on salivary proteomics identifies 65 proteins which change in expression with two distinctive expression patterns as a function of gestation age. The two distinct protein expression patterns deduce the interplay of relevant biological pathways. Our bioinformatics analyses have shown that the function of these processes is mediated through the 35 central regulatory proteins. Twelve of these have been validated through a separate subset of saliva samples in our cohort, in the targeted proteomics approach. The expression pattern of selected regulatory proteins in the MRM-based targeted data exhibits a similar expression pattern as observed in the SWATH-MS analysis. Our results show that the majority of differentially regulated proteins are associated with neutrophil degranulation, regulation of TLR by endogenous ligand, antimicrobial peptide function, platelet function regulation, and glucose metabolism. Here, we identified 35 seed proteins based on the degree and betweenness of interaction in the PPI network. Among them, 12 proteins have been selected in the MRM study as the majority belongs to the highest enriched pathway. Moreover, these proteins are also involved in more than one biological pathway except HSPA8 as indicated in Table 3. HSPA8 has been associated with the PPI network with the highest degree and betweenness (Table 4). We show that heat shock cognate 71 kDa protein (HSPA8/HSC70), protein S100-A8 (S100A8), protein S100-A9 (S100A9), cathepsin-D (CTSD), matrix metalloproteinase-9 (MMP9), fructose-bis-phosphate aldolase A (ALDOA), complement C3 (C3), lactotransferrin (LTF), myeloblastin (PRTN3), enolase-1 (Eno1) and galectin-3-binding protein (LGALS3BP) from cluster1, and synaptosomal-associated protein 23 (SNAP23) from cluster2 are the major 12 proteins which are associated with the above mentioned biological functions.

Heat stress cognate 70 is involved in the inflammatory signal pathways via extracellular interaction with TLR2/TLR4^24^ besides its chaperone function. We have detected more than two folds of increased expression of HSPA8 at V3 compared to V2. Similarly, TLR4 also recognizes calcium-binding S100 family members like S100A8 and S100A9^25,26^. In this study, we have not seen much change of S100A8/S100A9 expression in early pregnancy. However, more than three folds increased expression is observed at 26–28 weeks of gestation. It has been shown earlier that the high expression of S100A8 in serum in early pregnancy is associated with loss of pregnancy^27^. Protein S100A8, S100A9, and their heterodimer calprotectin (S100A8/S100A9) released by activated phagocytes have a role in inflammatory response by recruiting leukocytes and cytokine secretion^28,29^. A high abundance of calprotectin has been detected in amniotic fluid during intra amniotic infection, premature rupture of membranes and preterm labor^30^. Cathepsin-D promotes neutrophil apoptosis by activating caspase-8^31^. The deficiency of CTSD amplifies and prolongs neutrophilic inflammation *in vivo* and this pathway is crucial for the resolution of innate immune responses. We observed more than a two-fold increase in the CTSD level at V3 time point compared to V2. The plasma CTSD level is significantly lower in the first trimester compared to non-pregnant women and the expression level increases in the third trimester^32^.

During the advancement of pregnancy, continuous uterine tissue remodeling is required to accommodate the fetus^33^. The enlarged uterus in pregnancy undergoes dramatic changes during labor and the post-partum period^34^. The degradation and reorganization of extracellular matrix components (ECM) are the ongoing processes and it has been shown earlier that cytokines are involved in the production of MMP9 in human myometrium^34^. MMP9 degrades the wide range of ECM components^34,35^. An increase in MMP9 has been implicated in vasodilation, placentation, and uterine expansion during normal pregnancy. The decreased expression of MMP9 at the later stages of pregnancy may be associated with reduced vasodilation, increased vasoconstriction, and hypertensive pregnancy^36^. In the present study, we observe more than 1.5-folds increased expression of MMP9 at V3 time point compared to V1.

The tightly regulated expression of complement C3 in early and mid-pregnancy confirms its activation in a coordinated manner. C3 protein shows persistent expression throughout pregnancy in our data. Normal human pregnancy is associated with complement activation in plasma to combat pathogen attack^37^. An antimicrobial protein, lactotransferrin (LTF) also shows lower level expression at V2 time point compared to V1. However, with the progression of pregnancy, LTF concentration increases up to 2.0 folds at V3 time point compared to V1. LTF plays an important role in cervicovaginal infection by reducing cytokines level in cervicovaginal fluid^38^. The neutrophil-derived myeloblastin (PRTN3) plays an important role in re-establishing vascular integrity after leukocyte transmigration. During thrombotic and inflammatory events myeloblastin also protects endothelial cells from protease-activated receptor-1 induced permeability change^39^. More than 1.5 folds of increased expression of PRTN3 at a later stage of pregnancy might be induced neutrophils to secrete vascular integrity modulators and helps neutrophil transmigration^39^. SNAP23 has been involved in the secretion of gelatinase-rich tertiary granules from neutrophils and platelet α-granule release^40,41^. We observed high expression (>2 folds) of SNAP23 at V2 as compared to V1. Recent mice data have also confirmed that the deletion of the SNAP23 gene results in pre-implantation embryonic lethality^42^. The galectin-3-binding protein (LGLS3BP) shows consistent lower expression throughout pregnancy in our data. A recent study has shown that Galectin-3 induces preterm birth in the mouse model consequent to dental infection^43^. However, the precise role of LGALS3BP with galectin-3 in pregnancy is not elucidated.

Our data indicate that four proteins from cluster 1 enriched primarily to gluconeogenesis and glucose metabolism. Increased expression of ALDOA, enolase (Alfa and gamma; ENO1, and ENO2), malate dehydrogenase (MDH1) supports the enhanced gluconeogenesis at late pregnancy^44^. Increased expression of these proteins from early to mid-stage of pregnancy has been observed in our analysis. This confirms that enhance metabolism is an essential factor for fetal growth and development.

An important limitation of this study is the absence of a pre-pregnancy sample, that may have provided more in-depth insights on how pregnancy progresses from its conception to delivery. Similarly, the presence of samples beyond 29 weeks of pregnancy would have provided useful information on the protein trajectories till delivery. The comparative study of term versus preterm saliva as well as the plasma will provide a systematic variation of proteins that may be considered as potential biomarkers in pregnancy-related complications. The biobank of GARBH-Inicohort^45^ hosts saliva, plasma and high vaginal fluids samples for proteomics analysis. The biomarker identification in pregnancy-related complications like spontaneous preterm birth is underway using a nested case-control design.

Salivary samples are non-invasive and more accessible than other body fluids making them a better choice for the development of biomarkers of adverse pregnancy outcomes. Several studies have described the use of saliva as a diagnostic tool for many diseases^46-50^, but the application of saliva in pregnancy study needs further research. In conclusion, the present longitudinal salivary proteomics study provides a system-wide protein expression in normal pregnancy. The results suggested that 65 proteins were altered in expression with gestational age with two distinct clusters. Further functional enrichment analysis elucidates the involvement of these proteins in pregnancy-related biological processes. To the best of our knowledge, this is the first report on the longitudinal changes in salivary proteomics across term pregnancy. We believe the findings of this study may be useful for future studies to find either biomarker or mechanistic understanding of adverse pregnancy outcomes.

## Materials and Methods

### Study population

A cohort of pregnant women was initiated in mid-2015 at the district hospital in Gurugram, Haryana, India with the primary mandate to generate a risk-prediction algorithm for preterm birth based on multidimensional risk factors assessed during pregnancy. The profile (detailed objectives and methodology) of this cohort, recognized as the GARBH-Ini cohort is provided in an earlier publication^45^. Women are enrolled within 20 weeks of gestation and are followed at 4–5 time points during the antenatal period until delivery and once within 6 months of post-partum. Various bio-specimens are collected and ultrasound scans are performed at defined intervals as per protocol. This includes maternal saliva collected at enrolment (V1), 18–20 weeks (V2), and 26–29 weeks (V3) of gestation. The collection of saliva at delivery was avoided as the participants wouldn’t fulfill the preparatory criteria for collection as detailed below. Details of the ongoing collaborative interdisciplinary program (*GARBH-Ini)* is published elsewhere^45^.

#### Ethical consideration

Institutional Ethics Committees (Human Research) of Regional Centre for Biotechnology (RCB), Faridabad; Institutional Human Ethics Committee of Translational Health Science and Technology Institute (THSTI); Institutional Ethics Committee of Gurugram Civil Hospital (GCH) and Institutional Ethics Committee of Safdarjung Hospital (SJH)approved this study^45^. The detail ethical approval and consents were described earlier^45^. Briefly, eligible women were enrolled in the cohort after they gave their written informed consent. A broad consent was taken for analysis of biospecimens for research use after appropriate de-identification. All methods were performed in accordance with the relevant guidelines and regulations.

#### Selection of participants

A small subgroup of participants who had been enrolled before 14 completed weeks of gestation during April-October of any year of enrollment, delivered at term (37–40 weeks of gestational age) with singleton vaginal delivery, and had all three time-point samples were randomly selected for our stated objectives from the ongoing GARBH-Ini cohort. Those with pregnancy-related complications (such as pregnancy-induced hypertension, preeclampsia, eclampsia), associated co-morbidity (e.g. infective or metabolic) before or at any time during pregnancy, congenital anomalies, and multiple pregnancies were excluded. This ensured a homogenous group of women who had a normal term pregnancy. Of the 34 participants selected, 20 were considered as discovery cohort for SWATH-MS discovery proteomics study, and another 14 for verification of target proteins using multiple reaction monitoring (MRM) mass spectrometry.

### Sample collection, processing, and storage

The maternal saliva was collected from the enrolled participants and was processed in the research laboratory established at the hospital using standardized operating protocols that have been harmonized with other such global cohorts^45,51^. In brief, a non-stimulated saliva sample was collected in the morning prior to eating in a plastic container and 100 X halt protease and phosphatase inhibitors were added to a final concentration 1X. Saliva was immediately centrifuged at 10,000 g at 4 °C for 30 min. The supernatants were filtered through a 0.45-micron syringe filter and stored at −78 °C in the biorepository established for the study at the Translational Health Science and Technology Institute prior to mass spectrometry analysis. All the samples were retrieved just before the mass spectrometry experiment.

### Sample preparation for mass spectrometry

All samples were buffer exchanged five times with 100 mM ammonium bicarbonate (pH 8.0) through a 3 kDa MWCO membrane (Amicon Ultra, Millipore). Protein concentration was estimated with BCA protein assay kit and in-solution trypsin digestion was performed. Briefly, saliva samples were reduced with 5 mM DTT at 56 °C for 60 min and alkylated with 20 mM iodoacetamide in dark at 25 °C for 30 min. MS-grade trypsin (Pierce Thermo Fisher Scientific) was added with 20:1 protein to enzyme ratio and the digestion mixture was incubated at 37 °C for overnight. The pH of the solution was lowered to 2.0 with 1% formic acid to quench the activity of trypsin. The digested samples were evaporated to dryness and these samples were desalted with C18 fast-flow tips (Pierce Thermo Fisher Scientific). Desalted peptides were dried in vacuum and stored at −20 °C for further LC-MS/MS data acquisition. Before the acquisition, retention time calibration iRT peptides (Biognosys, Switzerland) were spiked to all the samples at a 1:10 ratio for retention time normalization^52^.

### Spectral library generation in Data-Dependent Acquisition (DDA)

Saliva specific peptide spectral library was created with pooled samples (N = 20) from all-time points V1, V2, and V3 in a combination of 1D-SDS-PAGE followed by in-gel digestion and in-solution digestion followed by basic reverse phase chromatography strategy (Fig. 1). SDS-PAGE was performed with 20 µg of pooled saliva protein and subsequently, in-gel digestion was performed. Basic reverse-phase chromatography was performed in the 1260 Infinity HPLC system which was equipped with an autosampler and fraction collector. A total of 600 µg of resulting peptides from pooled saliva were loaded onto C18 column (Agilent, 300 extend-C18; 3.5 µm; 2.1 × 150 mm) at oven temperature 40 °C which was previously equilibrated with solvent A (solvent A: 10 mM ammonium formate, pH 10; solvent B: 10 mM ammonium formate in 90% acetonitrile, pH 10). The peptides were eluted with the gradient from 2–50% of solvent B over 65 min total run at a flow rate of 500 µl/min. The eluted fractions were pooled, vacuum dried and desalted with C18 tips (Pierce). All fractions were resuspended in solvent C (composition: 2% (v/v) acetonitrile, 0.1% (v/v) formic acid in water) and analyzed in Sciex 5600^+^ Triple-TOF mass spectrometer which was coupled with ChromXP reversed-phase 3 μm C18-CL trap column (350 μm × 0.5 mm, 120 Å, Eksigent) and nanoViper C18 separation column (75 μm × 250 mm, 3 μm, 100 Å; Acclaim Pep Map, Thermo Scientific) in Eksigent nanoLC (Ultra 2D plus) system. The peptides were separated using a 90-min acetonitrile gradient from 5–50% of solvent D (composition: 98% (v/v) acetonitrile, 0.1% (v/v) formic acid) at a flow rate of 300 nl/min with column temperature of 40 °C. Data of each fraction was acquired in DDA with positive ionization mode. A maximum of 25 precursor ions with charge state 2–5 which surpass 120 cps per cycle was selected for fragmentation and each MS/MS spectrum was accumulated in high sensitivity mode. Each cycle consisted of 250 and 80–100 ms acquisition time for MS1 (m/z 350–1250 Da) and MS/MS (140–1800 m/z) scans respectively with a total cycle time of ~2.3 to 2.8 seconds. Dynamic exclusion was employed for 8–10 sec. Each fraction was analyzed majorly in duplicate. A blank of solvent C was run between each type of sample to reduce the carryover. Mass spectrometer calibration was performed at the start of each sample using a β-galactosidase digest standard.

### Data Independent Acquisition (DIA) for label-free peptide quantitation

To collect quantitative data each sample was injected in 3 replicates using identical LC conditions as DDA run. MS/MS data were acquired in SWATH mode and the instrument was configured as described by the previous study^53^. Briefly, in SWATH-MS mode, a set of 60 overlapping variable windows were constructed covering a mass range of 350–1250 m/z. The MS2 spectra were collected from 200–1800 m/z with the accumulation time of 60 ms in high sensitivity mode. Each SWATH‐MS cycle consisted of 100 ms of survey scan resulting in a total duty cycle of ~3.7 sec.

### Targeted mass spectrometry of selected proteins

Liquid chromatography-multiple reaction monitoring (LC-MRM) assay was performed on a QTRAP 6500^+^ mass spectrometer (Sciex) coupled with the LC system (Nexera). Saliva digested peptides (8 µg) were mixed with β- galactosidase (78 fmol) and iRT peptides (1:10) prior to sample load onto a C18 RP column (Agilent, 300 extend-C18; 3.5 µm; 2.1 × 150 mm). The gradient of acetonitrile was ranging from 5–50% of the total 60 min run at a flow rate of 0.3 ml/min. The binary mobile solvent system was used as follows; solvent E comprised 0.1% formic acid in the water, and solvent F included 100% acetonitrile with 0.1% formic acid. Spiked peptides were used for routine assessment of instrument and chromatographic performance. Skyline (v.4.1), SRM-Atlas (http://www.srmatlas.org/), and our spectral library were employed to select precursors of most intense y and b ions (transitions)^54,55^. Two transitions per precursors were traced for quantitation (Summarized in Supplementary Table S3). MRM data were acquired in the positive ion mode with an ion source temperature of 550 °C with the spray voltage of 5500 V. Scheduled MRM transition was performed using a detection time window of 60 sec. All MRM data were analyzed by MultiQuant (version 3.0.3, Sciex). The MQ4 integration algorithm within MultiQuant was utilized for peak integration. All MRM peaks were inspected manually to ensure correct peak detection and accurate integration. Two technical repeats were performed for each of the individual samples. The raw MRM peak area of two transitions was summed for each precursor. The average peak area values of each precursor from two technical repeats were transformed to log base 2, and demonstrated as a response of respective proteins with gestational age.

### Data analysis and availability

The IDA spectra were analyzed in Andromeda within the MaxQuant analysis software (version 1.5.4.1) with default settings^56,57^. IDA Search parameters were shown in Supplementary Table S2. Datasets were searched against the UniProt human reference protein database^58^(downloaded on July 2017) appended with iRT peptides fasta database (https://biognosys.com/). A false discovery rate (FDR) of 1% was imposed for peptide-spectrum matches (PSMs) and the target-decoy approach for protein identification. The saliva-specific ‘merged’ library (Supplementary Table S2) was built from the MaxQuant analysis file using Spectronaut (version 11) with inbuilt ID Picker algorithm^59,60^. All DIA datasets (.wiff files) were converted to HTRMS format and calibrated with retention time dimension using the saliva-specific ‘merged’ spectral library. Data were analyzed with Spectronaut 11 by default settings^61,62^. Both precursor and protein level Q value was set to 0.01. Global normalization was applied to correct a systematic variance in the LC-MS performance^63^. Spectronaut uses all precursors for the statistical test. However, only topN values are selected for the quantification. The protein quantitation and peptide quantities were calculated as a mean intensity within the XIC peak area of the respective fragment ions at MS2. The protein CVs were calculated based on the summed intensities of their respective peptides. The mass spectrometry proteomics data have been deposited to the ProteomeXchange Consortium via the PRIDE partner repository with the dataset identifier ‘PXD014800’.

### Statistical analysis

Clinical characteristics of the enrolled study group were summarized as median (IQR) for continuous variables, and percentages for categorical variables using Stata 15.1 software (StataCorp LP, College Station, TX, US). Data quality assessment for DIA data between technical replicates and within visit window V1, V2, and V3 peak intensity throughout LC run were log base2 transformed and Pearson correlation coefficient was computed using Graph Pad Prism (version 7). The statistical analysis for longitudinal changes of proteins with the function of Period of gestation (POG) was performed in R statistical language and environment (www.r-project.org)^64^. Protein abundance from the DIA dataset of all conditions (V1, V2, and V3) with 3 replicates for 20 samples were taken from the output file of Spectronaut and transformed to log base2 to improve normality. The proteins with unique identities were retained. The proteins which shared peptides and the peptides identified as contaminants were omitted from further analyses. Linear mixed-effects models were used to model the abundance of proteins as a function of gestational age using the *lme4* package^65^. The statistical significance of associations was estimated by comparing the models with and without gestational age by performing likelihood ratio tests using ANOVA function. Similarly, for MRM data mean peak area of each precursor was transformed to log base2 and linear mixed-effects models were applied. The changes in protein abundance across the pregnancy were considered significant if FDR adjusted p-value (q-value) was less than 0.1.

### Clustering, pathway enrichment, and protein-protein interaction (PPI) network analysis

The proteins that showed a significant change as a function of POG by the linear mixed-effects models were taken forward for clustering analysis. As our longitudinal data had short time series and unevenly spaced samples we used short time-series distance, a method specifically designed to address these challenges followed by hierarchical clustering^66,67^. Plots to show the pattern of longitudinal changes in protein abundance were shown for visit windows 1, 2 and 3. Reactome database was queried for the pathway enrichment analysis using an R-package, ReactomePA^68^. The protein-protein interaction (PPI) network was built by NetworkAnalyst with the IMEX interactome database^69^. The node parameters degree and betweenness were considered for the identification of central regulators and were determined through NetworkAnalyst. “Minimum network” of the modulated proteins was constructed in a force atlas layout format. Subnetwork with at least 3 nodes was constructed. The expression of the nodes was symbolized by their colors.

## Supplementary information

Supplementary information.
