# Supplementary material for: Salivary proteome signatures in the early and middle stages of human pregnancy with term birth outcome
Source: Sci Rep. 2020 May 15;10:8022. doi: 10.1038/s41598-020-64483-6 (PMC7229191; doi:10.1038/s41598-020-64483-6)
Supplement: Supplementary file 1 — Supplementary information. [file 41598_2020_64483_MOESM1_ESM.docx]

**Supplementary information**

**Salivary proteome signatures in the early and middle stages of human pregnancy with term birth outcome**

Amit Kumar Dey^1§^, Bhoj Kumar^1§^, Abhishek Kumar Singh^1^, Prakash Ranjan^1^, Ramachandran Thiruvengadam^2^, Bapu Koundinya Desiraju^2^, Pallavi Kshetrapal^2^, Nitya Wadhwa^2^, Shinjini Bhatnagar^2*^, Faraz Rashid^3^, Dipankar Malakar^3^, Dinakar M Salunke^1,4*^, Tushar Kanti Maiti^1*^and GARBH-Ini Study Group**

^1^Regional Centre for Biotechnology, NCR Biotech Science Cluster, Faridabad 121001, INDIA

^2^Translational Health Science and Technology Institute, NCR Biotech Science Cluster, Faridabad 121001, INDIA.

^3^Sciex, 121 UdyogVihar, Gurgaon, Haryana 122015, India.

^4^International Centre for Genetic Engineering and Biotechnology, Aruna Asaf Ali Marg, New Delhi, 110067, INDIA

**Address correspondence to:**

Dr. Tushar Kanti Maiti,

Regional Centre for Biotechnology, NCR Biotech Science Cluster, Faridabad 121001, India.

Email: [tkmaiti@rcb.res.in](mailto:tkmaiti@rcb.res.in)

Dr. Dinakar M Salunke

International Centre for Genetic Engineering and Biotechnology, Aruna Asaf Ali Marg, New Delhi, 110067, India

Email: [dinakar.salunke55@gmail.com](mailto:dinakar.salunke55@gmail.com)

Dr. Shinjini Bhatnagar

Translational Health Science and Technology Institute, NCR Biotech Science Cluster, Faridabad 121001, India

Email: [shinjini.bhatnagar@thsti.res.in](mailto:shinjini.bhatnagar@thsti.res.in)

** Other members of the GARBH-Ini Study Group:

Bhabatosh Das^2^, Sumit Misra^2^, Balakrish G. Nair^2^, Uma Chandra Mouli Natchu^2^, Satyajit Rath^2,5^, Kanika Sachdeva^2^, Shailaja Sopory^2^, Amanpreet Singh^2^, Dharmendra Sharma^2^, Vineeta Bal^2,5^, Arindam Maitra^6^, Partha P. Majumder^6^, Monika Bahl^7^, Sunita Sharma^8^, Umesh Mehta^8^, Brahmdeep Sindhu^8^, Sugandha Arya^9^, Rekha Bharti^9^, Harish Chellani^9^, Pratima Mittal^9^, Siddarth Ramji^10^, Reva Tripathi^10,11^, Anju Garg^10^, Ashok Khurana^12^, Smriti Hari^13^, Yashdeep Gupta^13^, Nikhil Tandon^13^, Rakesh Gupta^14^

^5^Indian Institute of Science Education and Research, Pune, Maharashtra, India

^6^National Institute of Biomedical Genomics, Kalyani, West Bengal, India

^7^Clinical Development Services Agency, Translational Health Science and Technology Institute, NCR Biotech Cluster, Faridabad, Delhi NCR, India

^8^Gurugram Civil Hospital, Haryana, India

^9^Safdarjung Hospital, New Delhi, India

^10^Maulana Azad Medical College, New Delhi, India

^11^Hamdard Institute of Medical Sciences and Research, Jamia Hamdard University, New Delhi, India

^12^The Ultrasound Lab, Defence Colony, New Delhi, India

^13^All India Institute of Medical Sciences, New Delhi, India

^14^Government of Haryana, India

**Table legends:**

**Table1**: **Clinical and demographic characteristics of the study population**. Data are presented as number (percentage) for categorical variables or median (Inter Quartile Range (IQR)) for continuous variables.

**Table 2**: **List of proteins modulates with the function of gestational age**. Fold change (FC) stands for the ratio in mean protein abundance between visit windows (V1, V2, and V3). Protein names with UniProt Id, gene name, and cluster membership are provided in the table. Proteins are considered to be significant when q values < 0.1 from linear mixed-effects models.

**Table 3**: **Enriched biological pathways linked with the proteins that are changed as a function of gestational age.** Adjusted p values (˂ 0.05) from the Benjamini and Hochberg method are given along with all biological pathways.

**Table 4**: **Degree and betweenness centrality values of the central regulators.** Gene name along with UniProt id is shown.

**

**

**Figure S1:** **Global enrichment of proteins identified in the spectral library**. Enrichment with 758 proteins was performed using the Reactome database in NetworkAnalyst (<https://www.networkanalyst.ca/>), and are shown in a force atlas layout format. A total of 540 proteins were enriched in 57 pathways (FDR< 0.05) and 46 pathways are shown in the figure. Nodes represent enriched pathways and edges between two nodes represent interactions between them. The colors denote the significance, red to blue indicates higher to lower significant pathways. The intensity of the colors signifies the significance level.


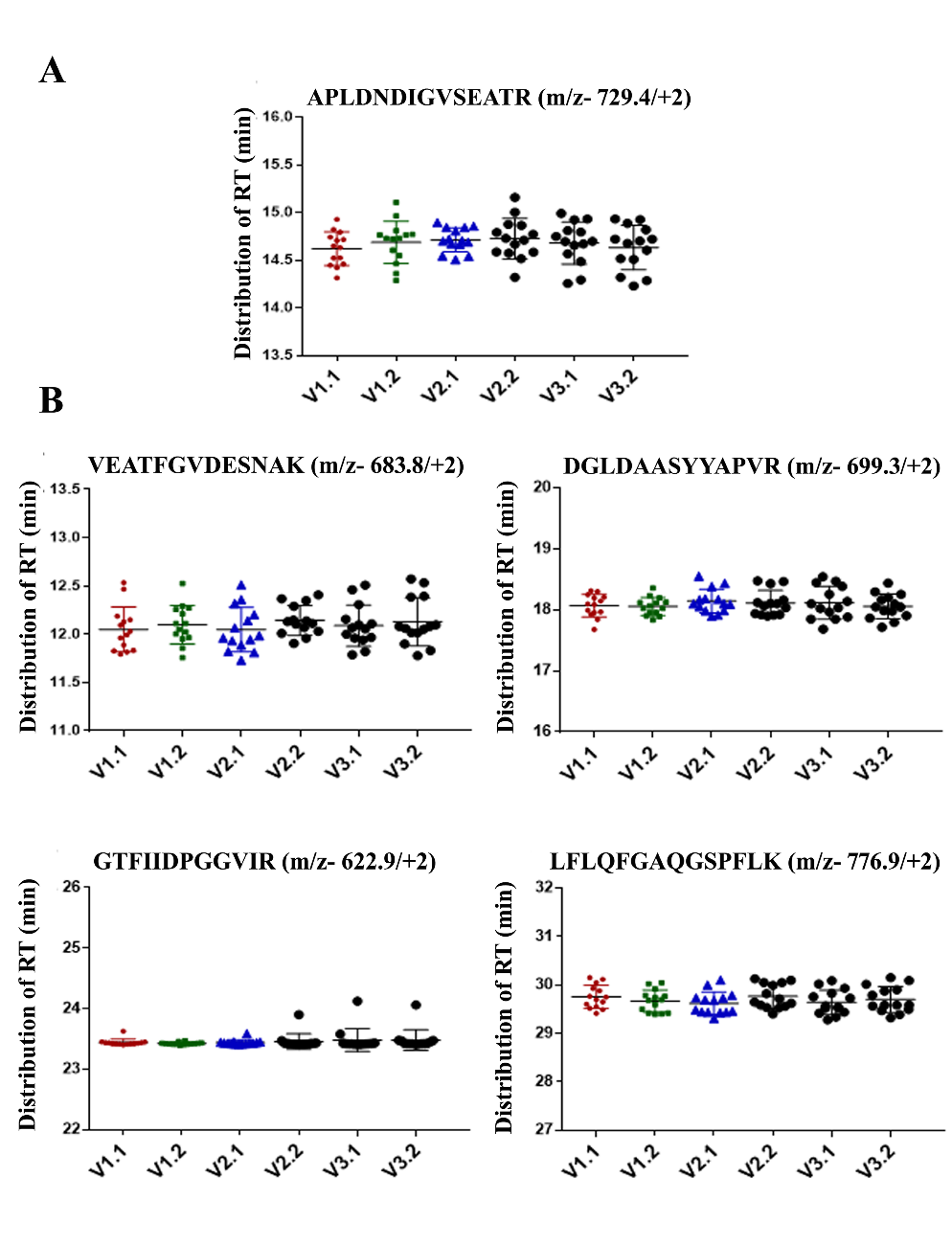


**Figure S2:** **Reproducibility of retention time of precursors in MRM spiked in the saliva sample**. β-galactosidase (A) and of iRT (B) within 14 saliva sample was monitored and is shown in the figure. Transitions have consistent retention time with a coefficient of variation of less than 2.0%.

**Table S1**: **List of significant pathways (FDR< 0.05) enriched with 758 proteins of spectral library**.

| **Pathway** | **Total** | **Expected** | **Hits** | **P.Value** | **FDR** |
| --- | --- | --- | --- | --- | --- |
| 1. Amyloids | 84 | 3.54 | 33 | 0.000 | 0.000 |
| 1. Platelet degranulation | 89 | 3.75 | 29 | 0.000 | 0.000 |
| 1. Packaging Of Telomere Ends | 48 | 2.02 | 22 | 0.000 | 0.000 |
| 1. Response to elevated platelet cytosolic Ca2+ | 94 | 3.96 | 29 | 0.000 | 0.000 |
| 1. Meiotic Synapsis | 76 | 3.2 | 24 | 0.000 | 0.000 |
| 1. Meiotic Recombination | 84 | 3.54 | 25 | 0.000 | 0.000 |
| 1. Telomere Maintenance | 72 | 3.03 | 23 | 0.000 | 0.000 |
| 1. Deposition of New CENPA-containing Nucleosomes at the Centromere | 65 | 2.74 | 21 | 0.000 | 0.000 |
| 1. Nucleosome assembly | 65 | 2.74 | 21 | 0.000 | 0.000 |
| 1. Meiosis | 117 | 4.92 | 27 | 0.000 | 0.000 |
| 1. Platelet activation, signaling and aggregation | 220 | 9.26 | 37 | 0.000 | 0.000 |
| 1. Glucose metabolism | 70 | 2.95 | 21 | 0.000 | 0.000 |
| 1. Chromosome Maintenance | 124 | 5.22 | 25 | 0.000 | 0.000 |
| 1. Hemostasis | 511 | 21.5 | 53 | 0.000 | 0.000 |
| 1. Gluconeogenesis | 35 | 1.47 | 13 | 0.000 | 0.000 |
| 1. Metabolism of carbohydrates | 258 | 10.9 | 33 | 0.000 | 0.000 |
| 1. Smooth Muscle Contraction | 25 | 1.05 | 10 | 0.000 | 0.000 |
| 1. Scavenging of Heme from Plasma | 15 | 0.631 | 8 | 0.000 | 0.000 |
| 1. Binding and Uptake of Ligands by Scavenger Receptors | 15 | 0.631 | 8 | 0.000 | 0.000 |
| 1. Muscle contraction | 52 | 2.19 | 13 | 0.000 | 0.000 |
| 1. Glycolysis | 30 | 1.26 | 10 | 0.000 | 0.000 |
| 1. Apoptosis | 158 | 6.65 | 22 | 0.000 | 0.000 |
| 1. Formation of Fibrin Clot (Clotting Cascade) | 29 | 1.22 | 8 | 0.000 | 0.001 |
| 1. Uptake of Oxygen and Release of Carbon Dioxide by Erythrocytes | 10 | 0.421 | 5 | 0.000 | 0.001 |
| 1. Uptake of Carbon Dioxide and Release of Oxygen by Erythrocytes | 10 | 0.421 | 5 | 0.000 | 0.001 |
| 1. O2/CO2 exchange in erythrocytes | 10 | 0.421 | 5 | 0.000 | 0.001 |
| 1. Activation of BAD and translocation to mitochondria | 17 | 0.715 | 6 | 0.000 | 0.002 |
| 1. Common Pathway | 11 | 0.463 | 5 | 0.000 | 0.002 |
| 1. Advanced glycosylation endproduct receptor signaling | 12 | 0.505 | 5 | 0.000 | 0.004 |
| 1. Synthesis and interconversion of nucleotide di- and triphosphates | 20 | 0.842 | 6 | 0.000 | 0.006 |
| 1. Metabolism of nucleotides | 81 | 3.41 | 12 | 0.000 | 0.006 |
| 1. Metabolism | 1490 | 62.6 | 89 | 0.000 | 0.006 |
| 1. Glycogen synthesis | 8 | 0.337 | 4 | 0.000 | 0.008 |
| 1. Regulation of actin dynamics for phagocytic cup formation | 62 | 2.61 | 10 | 0.000 | 0.009 |
| 1. Striated Muscle Contraction | 31 | 1.3 | 7 | 0.000 | 0.010 |
| 1. ER-Phagosome pathway | 63 | 2.65 | 10 | 0.000 | 0.010 |
| 1. GRB2:SOS provides linkage to MAPK signaling for Intergrins | 15 | 0.631 | 5 | 0.000 | 0.010 |
| 1. p130Cas linkage to MAPK signaling for integrins | 15 | 0.631 | 5 | 0.000 | 0.010 |
| 1. Pentose phosphate pathway (hexose monophosphate shunt) | 9 | 0.379 | 4 | 0.000 | 0.012 |
| 1. Destabilization of mRNA by AUF1 (hnRNP D0) | 54 | 2.27 | 9 | 0.000 | 0.013 |
| 1. Activation of BH3-only proteins | 24 | 1.01 | 6 | 0.000 | 0.013 |
| 1. Antigen processing-Cross presentation | 78 | 3.28 | 11 | 0.000 | 0.013 |
| 1. Apoptotic execution phase | 57 | 2.4 | 9 | 0.001 | 0.018 |
| 1. Glycogen breakdown (glycogenolysis) | 18 | 0.758 | 5 | 0.001 | 0.022 |
| 1. Calnexin/calreticulin cycle | 11 | 0.463 | 4 | 0.001 | 0.025 |
| 1. Fcgamma receptor (FCGR) dependent phagocytosis | 86 | 3.62 | 11 | 0.001 | 0.027 |
| 1. Cell-extracellular matrix interactions | 19 | 0.8 | 5 | 0.001 | 0.027 |
| 1. Regulation of mRNA Stability by Proteins that Bind AU-rich Elements | 88 | 3.7 | 11 | 0.001 | 0.031 |
| 1. Cell Cycle | 508 | 21.4 | 36 | 0.001 | 0.036 |
| 1. Degradation of the extracellular matrix | 77 | 3.24 | 10 | 0.001 | 0.038 |
| 1. Disease | 945 | 39.8 | 58 | 0.002 | 0.041 |
| 1. N-glycan trimming in the ER and Calnexin/Calreticulin cycle | 13 | 0.547 | 4 | 0.002 | 0.041 |
| 1. Apoptosis induced DNA fragmentation | 13 | 0.547 | 4 | 0.002 | 0.041 |
| 1. Activation of DNA fragmentation factor | 13 | 0.547 | 4 | 0.002 | 0.041 |
| 1. Regulation of the Fanconi anemia pathway | 13 | 0.547 | 4 | 0.002 | 0.041 |
| 1. Insulin effects increased synthesis of Xylulose-5-Phosphate | 2 | 0.0842 | 2 | 0.002 | 0.043 |
| 1. Nicotinamide salvaging | 2 | 0.0842 | 2 | 0.002 | 0.043 |

**Table S2: Spectral library overview**. Four different combinations of cleavage enzyme were used during the analysis in Maxquant, for example with trypsin and trypsin/P both specific and semispecific was used separately. Other parameters as shown table are same in each analysis. Four independent analysis was merged for the final output of spectral library.

| **Software** | **Spectral Library** | **Precursors** | **Proteins** | **Protein groups** | **Search Criteria** |
| --- | --- | --- | --- | --- | --- |
| Maxquant | S_trypsin_specific | 3336 | 576 (687) | 512 (530) | Trypsin and Trypsin/P separately as cleavage enzyme; Up to two miss cleavage; Carbamidomethyl (C), Oxidation (M), Acetyl (Protein N-term) as a variable modification |
|  | S_trypsin_semispecific | 2772 | 392 (453) | 321 (337) |  |
|  | S_trypsin/P_specific | 3278 | 550 (660) | 500 (517) |  |
|  | S_trypsin/P_semispecific | 2737 | 380 (440) | 316 (333) |  |
| Spectronaut | S_merged | 4534 | 594 (758) | 530 (616) |  |

**Table S3: MRM parameters of the precursors used for relative quantitation of 12 proteins in saliva**.

| **Uniprot Id** | **Gene Name** | **Precursor sequence** | **Q1_m/z** | **Q1_charge** | **Q3_m/z** | **Q3_charge** | **Ion** |
| --- | --- | --- | --- | --- | --- | --- | --- |
| P04075 | ALDOA | ADDGRPFPQVIK | 448.2 | 3 | 578.8 | 2 | y10 |
|  |  | ADDGRPFPQVIK | 448.2 | 3 | 636.3 | 2 | y11 |
|  |  | GILAADESTGSIAK | 666.9 | 2 | 907.4 | 1 | y9 |
|  |  | GILAADESTGSIAK | 666.9 | 2 | 774.3 | 1 | y8 |
|  |  | IVAPGKGILAADESTGSIAK | 633.4 | 3 | 907.4 | 1 | y9 |
|  |  | IVAPGKGILAADESTGSIAK | 633.4 | 3 | 663.4 | 1 | y7 |
| P01024 | C3 | IPIEDGSGEVVLSR | 735.9 | 2 | 903.5 | 1 | y9 |
|  |  | IPIEDGSGEVVLSR | 735.9 | 2 | 846.5 | 1 | y8 |
|  |  | ISLPESLK | 443.8 | 2 | 573.3 | 1 | y5 |
|  |  | ISLPESLK | 443.8 | 2 | 773.4 | 1 | y7 |
|  |  | LVAYYTLIGASGQR | 504.6 | 3 | 575.3 | 1 | y6 |
|  |  | LVAYYTLIGASGQR | 504.6 | 3 | 688.4 | 1 | y7 |
| P05109 | S100A8 | ALNSIIDVYHK | 636.9 | 2 | 661.3 | 1 | y5 |
|  |  | ALNSIIDVYHK | 636.9 | 2 | 447.2 | 1 | y3 |
|  |  | NSIIDVYHK | 544.8 | 2 | 447.2 | 1 | y3 |
|  |  | NSIIDVYHK | 544.8 | 2 | 774.4 | 1 | y6 |
|  |  | ALNSIIDVYHK | 424.9 | 3 | 661.3 | 1 | y5 |
|  |  | ALNSIIDVYHK | 424.9 | 3 | 774.4 | 1 | y6 |
| P06702 | S100A9 | LGHPDTLNQGEFK | 485.9 | 3 | 480.3 | 1 | y4 |
|  |  | LGHPDTLNQGEFK | 485.9 | 3 | 621.3 | 1 | b6 |
|  |  | VIEHIMEDLDTNADK | 581.6 | 3 | 663.3 | 1 | y6 |
|  |  | VIEHIMEDLDTNADK | 581.6 | 3 | 548.3 | 1 | y5 |
|  |  | ETIINTFHQYSVK | 790.4 | 2 | 624.3 | 1 | y5 |
|  |  | ETIINTFHQYSVK | 790.4 | 2 | 1123.6 | 1 | y9 |
| P02788 | LTF | DGAGDVAFIR | 510.8 | 2 | 777.4 | 1 | y7 |
|  |  | DGAGDVAFIR | 510.8 | 2 | 506.3 | 1 | y4 |
|  |  | GGSFQLNELQGLK | 695.9 | 2 | 801.5 | 1 | y7 |
|  |  | GGSFQLNELQGLK | 695.9 | 2 | 317.2 | 1 | y3 |
|  |  | FQLFGSPSGQK | 598.3 | 2 | 807.4 | 1 | y8 |
|  |  | FQLFGSPSGQK | 598.3 | 2 | 516.3 | 1 | y5 |
| P07339 | CTSD | FDGILGMAYPR | 620.3 | 2 | 694.3 | 1 | y6 |
|  |  | FDGILGMAYPR | 620.3 | 2 | 807.4 | 1 | y7 |
|  |  | QVFGEATKQPGITFIAAK | 636.0 | 3 | 415.2 | 1 | b4 |
|  |  | QVFGEATKQPGITFIAAK | 636.0 | 3 | 650.4 | 1 | y6 |
|  |  | LSPEDYTLK | 533.3 | 2 | 639.3 | 1 | y5 |
|  |  | LSPEDYTLK | 533.3 | 2 | 361.2 | 1 | y3 |
| P14780 | MMP9 | LGLGADVAQVTGALR | 720.9 | 2 | 744.3 | 1 | y7 |
|  |  | LGLGADVAQVTGALR | 720.9 | 2 | 517.3 | 1 | y5 |
|  |  | AVIDDAFAR | 489.3 | 2 | 694.3 | 1 | y6 |
|  |  | AVIDDAFAR | 489.3 | 2 | 579.3 | 1 | y5 |
|  |  | QLSLPETGELDSATLK | 851.5 | 2 | 1034.5 | 1 | y10 |
|  |  | QLSLPETGELDSATLK | 851.5 | 2 | 311.2 | 1 | b3 |
| P24158 | PRTN3 | LVNVVLGAHNVR | 430.9 | 3 | 766.4 | 1 | y7 |
|  |  | LVNVVLGAHNVR | 430.9 | 3 | 865.5 | 1 | y8 |
|  |  | VALYVDWIR | 567.8 | 2 | 688.4 | 1 | y5 |
|  |  | VALYVDWIR | 567.8 | 2 | 474.3 | 1 | y3 |
|  |  | LFPDFFTR | 521.8 | 2 | 570.3 | 1 | y4 |
|  |  | LFPDFFTR | 521.8 | 2 | 685.3 | 1 | y5 |
| P06733 | ENO1 | YISPDQLADLYK | 713.4 | 2 | 1149.6 | 1 | y10 |
|  |  | YISPDQLADLYK | 713.4 | 2 | 609.3 | 1 | y5 |
|  |  | IGAEVYHNLK | 381.9 | 3 | 515.8 | 2 | y9 |
|  |  | IGAEVYHNLK | 381.9 | 3 | 674.4 | 1 | y5 |
|  |  | AAVPSGASTGIYEALELR | 903.0 | 2 | 1063.6 | 1 | y9 |
|  |  | AAVPSGASTGIYEALELR | 903.0 | 2 | 893.5 | 1 | y7 |
| Q08380 | LGALS3BP | IYTSPTWSAFVTDSSWSAR | 1081.5 | 2 | 1313.6 | 1 | y12 |
|  |  | IYTSPTWSAFVTDSSWSAR | 1081.5 | 2 | 693.3 | 1 | y6 |
|  |  | YSSDYFQAPSDYR | 799.8 | 2 | 708.3 | 1 | y6 |
|  |  | YSSDYFQAPSDYR | 799.8 | 2 | 836.4 | 1 | y7 |
| O00161 | SNAP23 | [+42]M[+16]DNLSSEEIQQR | 754.3 | 2 | 673.4 | 1 | y5 |
|  |  | [+42]M[+16]DNLSSEEIQQR | 754.3 | 2 | 976.5 | 1 | y8 |
|  |  | TLTELNKC[+57]C[+57]GLC[+57]VC[+57]PCNR | 733.3 | 3 | 335.2 | 3 | y8 |
|  |  | TLTELNKC[+57]C[+57]GLC[+57]VC[+57]PCNR | 733.3 | 3 | 560.8 | 2 | b9 |
| P11142 | HSPA8 | IINEPTAAAIAYGLDK | 830.5 | 2 | 921.5 | 1 | y9 |
|  |  | IINEPTAAAIAYGLDK | 830.5 | 2 | 850.5 | 1 | y8 |
|  |  | FEELNADLFR | 627.3 | 2 | 848.5 | 1 | y7 |
|  |  | FEELNADLFR | 627.3 | 2 | 735.4 | 1 | y6 |
|  |  | SINPDEAVAYGAAVQAAILSGDK | 754.1 | 3 | 519.3 | 1 | y5 |
|  |  | SINPDEAVAYGAAVQAAILSGDK | 754.1 | 3 | 406.2 | 1 | y4 |

**Table S4: List of 12 targeted proteins from MRM analysis**. The log2 peak area at visit 1,2, and 3 was calculated. Fold change (FC) stands for the ratio in protein abundance between visits (V1, V2, and V3). UniProt Id and gene name are provided in the table. Proteins are considered to be significant when q values < 0.1 from linear mixed-effect models.

| **Uniprot Id** | **Gene Name** | **Precursor sequence** | **Transitions** | **FC (V2/V1)** | **FC (V3/V1)** | **FC (V3/V2)** | **p_values** | **q_values** |
| --- | --- | --- | --- | --- | --- | --- | --- | --- |
| P04075 | ALDOA | ADDGRPFPQVIK | 448.2/ 578.8; 636.3 | 1.69 | 2.15 | 1.27 | 0.000 | 0.000 |
|  |  | GILAADESTGSIAK | 666.9/ 907.4; 774.3 | 1.35 | 1.96 | 1.45 | 0.000 | 0.000 |
|  |  | IVAPGKGILAADESTGSIAK | 633.4/ 907.4; 663.4 | 1.20 | 2.16 | 1.79 | 0.000 | 0.000 |
| P01024 | C3 | IPIEDGSGEVVLSR | 735.9/ 903.5; 846.5 | 1.31 | 2.21 | 1.69 | 0.000 | 0.000 |
|  |  | ISLPESLK | 443.8/ 573.3; 773.4 | 1.16 | 2.41 | 2.09 | 0.000 | 0.000 |
|  |  | LVAYYTLIGASGQR | 504.6/ 575.3; 688.4 | 1.21 | 1.77 | 1.46 | 0.000 | 0.000 |
| P05109 | S100A8 | ALNSIIDVYHK | 636.9/ 661.3; 447.2 | 1.58 | 2.92 | 1.85 | 0.000 | 0.000 |
|  |  | NSIIDVYHK | 544.8/ 447.2; 774.4 | 1.36 | 2.84 | 2.09 | 0.000 | 0.000 |
|  |  | ALNSIIDVYHK | 424.9/ 661.3; 774.4 | 1.39 | 3.17 | 2.27 | 0.000 | 0.000 |
| P06702 | S100A9 | LGHPDTLNQGEFK | 485.9/ 480.3; 621.3 | 1.07 | 1.87 | 1.75 | 0.000 | 0.000 |
|  |  | VIEHIMEDLDTNADK | 581.6/ 663.3; 548.3 | 1.31 | 2.10 | 1.61 | 0.000 | 0.000 |
|  |  | ETIINTFHQYSVK | 790.4/ 624.3; 1123.6 | 1.50 | 3.05 | 2.04 | 0.000 | 0.000 |
| P02788 | LTF | DGAGDVAFIR | 510.8/ 777.4; 506.3 | 0.98 | 2.13 | 2.17 | 0.000 | 0.000 |
|  |  | GGSFQLNELQGLK | 695.9/ 801.5; 317.2 | 1.19 | 1.95 | 1.64 | 0.000 | 0.000 |
|  |  | FQLFGSPSGQK | 598.3/ 807.4; 516.3 | 1.07 | 1.81 | 1.69 | 0.000 | 0.000 |
| P07339 | CTSD | FDGILGMAYPR | 620.3/ 694.3; 807.4 | 0.92 | 1.71 | 1.86 | 0.000 | 0.000 |
|  |  | QVFGEATKQPGITFIAAK | 636/ 415.2; 650.4 | 1.11 | 2.15 | 1.94 | 0.000 | 0.000 |
|  |  | LSPEDYTLK | 533.3/ 639.3; 361.2 | 1.14 | 2.09 | 1.84 | 0.000 | 0.000 |
| P14780 | MMP9 | LGLGADVAQVTGALR | 720.9/ 744.3; 517.3 | 1.21 | 2.16 | 1.78 | 0.000 | 0.000 |
|  |  | AVIDDAFAR | 489.3/ 694.3; 579.3 | 1.29 | 2.12 | 1.64 | 0.000 | 0.000 |
|  |  | QLSLPETGELDSATLK | 851.5/ 1034.5; 311.2 | 1.15 | 2.28 | 1.99 | 0.000 | 0.000 |
| P24158 | PRTN3 | LVNVVLGAHNVR | 430.9/ 766.4; 865.5 | 1.68 | 2.70 | 1.60 | 0.000 | 0.000 |
|  |  | VALYVDWIR | 567.8/ 688.4; 474.3 | 1.05 | 1.91 | 1.81 | 0.000 | 0.000 |
|  |  | LFPDFFTR | 521.8/ 570.3; 685.3 | 1.19 | 1.94 | 1.63 | 0.000 | 0.000 |
| P06733 | ENO1 | YISPDQLADLYK | 713.4/ 1149.6; 609.3 | 1.35 | 1.81 | 1.34 | 0.000 | 0.000 |
|  |  | IGAEVYHNLK | 381.9/ 515.8; 674.4 | 1.15 | 1.74 | 1.51 | 0.000 | 0.000 |
|  |  | AAVPSGASTGIYEALELR | 903/ 1063.6; 893.5 | 1.41 | 1.95 | 1.38 | 0.000 | 0.000 |
| Q08380 | LGALS3BP | IYTSPTWSAFVTDSSWSAR | 1081.5/ 1313.6; 693.3 | 0.89 | 1.17 | 1.30 | 0.039 | 0.040 |
|  |  | YSSDYFQAPSDYR | 799.8/ 708.3; 836.4 | 0.99 | 1.31 | 1.33 | 0.018 | 0.019 |
| O00161 | SNAP23 | [+42]M[+16]DNLSSEEIQQR | 754.3/ 673.4; 976.5 | 1.36 | 1.54 | 1.13 | 0.000 | 0.000 |
|  |  | TLTELNKC[+57]C[+57]GLC[+57]VC[+57]PCNR | 733.3/ 335.2; 560.8 | 1.59 | 1.78 | 1.12 | 0.000 | 0.000 |
| P11142 | HSPA8 | IINEPTAAAIAYGLDK | 830.5/ 921.5; 850.5 | 0.79 | 1.28 | 1.63 | 0.000 | 0.000 |
|  |  | FEELNADLFR | 627.3/ 848.5; 735.4 | 1.07 | 2.01 | 1.87 | 0.000 | 0.000 |
|  |  | SINPDEAVAYGAAVQAAILSGDK | 754.1/ 519.3; 406.2 | 1.18 | 1.79 | 1.51 | 0.001 | 0.001 |
